# Working fluid selection for the geothermal-solar hybrid cycle at Olkaria II power plant in Kenya

**DOI:** 10.1016/j.heliyon.2022.e12762

**Published:** 2023-01-03

**Authors:** Hofni Dionisius Venomhata Venomhata, Peter Obara Oketch, Benson Baari Gathitu, Paul Chisale

**Affiliations:** a Department of Mechanical Engineering, Pan African University Institute for Basic Sciences, Technology and Innovation, P. O. Box 62000-00200, Nairobi, Kenya; b Department of Mechanical Engineering, Jomo Kenyatta University of Agriculture and Technology, P. O. Box 62000–00200, Nairobi, Kenya; c Department of Agricultural and Biosystems Engineering, Jomo Kenyatta University of Agriculture and Technology, P. O. Box 62000–00200, Nairobi, Kenya; d Department of Mechanical Engineering, The Copperbelt University, 4662 Jambo Drive, Riverside Campus, P. O. Box 21692, Kitwe, Zambia

**Keywords:** Flash hybrid, Global warming, Working fluids, Ozone, Therminol VP-1, Organic rankine cycle

## Abstract

At Olkaria II power plant located in Kenya, the brine from the separator is re-injected back into the ground. This study analyzes a flash geothermal-solar hybrid cycle using the working fluids n-butane, n-pentane, cyclopentane, hexamethyldisiloxane and toluene in order to utilize the brine. The studied working fluids have no potential to deplete the ozone or cause global warming. The working fluids were analyzed in terms of net power output and pump power required. This was done by heating Therminol VP-1 fluid to a maximum temperature of 400 °C using the parabolic trough solar collector and varying the working fluids mass flow rate, Therminol VP-1 flow rate and pressure, and the brine pressure and flow rate. They were varied to investigate their effect on the power output of the Organic Rankine Cycle. The working fluid with higher net power output was selected as a suitable working fluid for the hybrid cycle. The selected working fluid was used to determine the average hourly power output in each month of the hybrid system using the irradiance of Olkaria II power station. N-butane showed the best results in net power output hence it was selected as a suitable working fluid as compared to the other working fluids. When the hybrid system was analyzed using n-butane to obtain the hourly average power output in each month, the results revealed that the month of February has the highest hourly average power output where the Steam Turbine has an average hourly power output of 10.48 MW and Binary Turbine net power output of 18.56 MW. The minimum average hourly power output was obtained in the month of June where the Steam Turbine has an average hourly power output of 9.08 MW and Binary Turbine net power output of 16.88 MW.

## 1 Introduction

**Figure 1:**
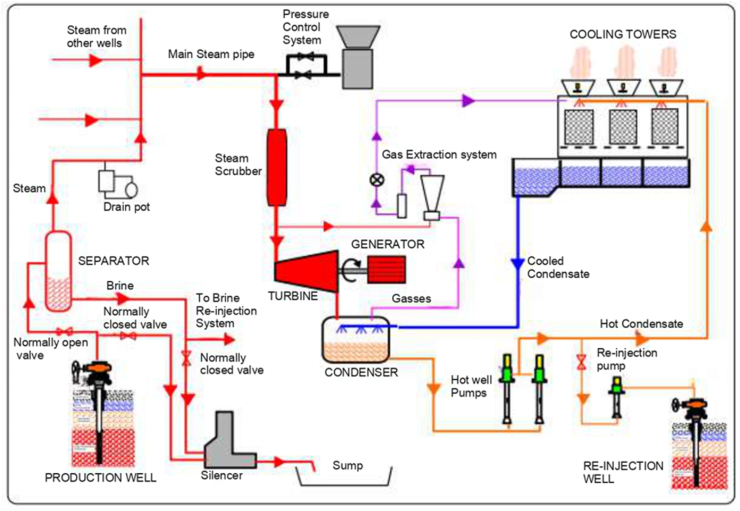
Olkaria II Power plant process diagram [4].

Among the sources of renewable energy, geothermal has the potential to resolve the energy demand in countries with geothermal reserves. Geothermal energy provides innumerable benefits over other sources of energy since is not affected by climatic variability [1,2]. Considering Africa, Kenya is the leading in geothermal energy with an installed capacity of 863 MW. The resources with geothermal in Kenya are found within the Rift Valley and has a potential estimated between 7 GW and 10 GW distributed among 14 potential sites. One of the geothermal power plants is known as Olkaria II power station that has the power generation capacity of 105 MW. The process flow diagram for Olkaria II power station is as illustrated in Figure 1. The geothermal steam from the production well is routed to the separator, where the steam is admitted to the steam turbine for power generation. The low temperature geothermal brine from the separator is re-injected back to the ground. According to Mburu [3], 100 MWt of energy can be extracted from the brine produced by the Olkaria II power plant.

For effective utilization of the geothermal brine, here, a case is considered where the parabolic-trough solar collector is providing additional heat to the brine that is being re-injected back into the ground. The brine is heated to a higher temperature, which allows flashing to occur and produce more energy. Solar energy is dependent on the weather and it is not available at night, on cloudy days, or on dusty days. However, geothermal energy is a consistent source of power that is unaffected by weather, making it a good alternative for achieving the needed energy output when combined with solar energy. When solar and geothermal are integrated it will help obtain the optimum power output for example during the day at peak hours it will be able to produce more electricity to meet people's energy demand. A hybrid of geothermal and solar has a longer life span than a standalone power plant since the hybrid cycle uses less brine [5].

The hybrid system is more affordable because it does not have the cost of exploration which involves well drilling. The exploration was performed already at the setup of Olkaria II power plant. Some of the environment and economic benefits on geothermal and solar hybrid system are as indicated in some studies below. For a geothermal power plant that uses a parabolic trough solar collector size of $250/m^2^, the electricity cost is between $0.19 and $0.25 per kWh at an interest rate of 8.8% [6]. Bonyadi et al. [5] performed an analysis on solar rankine cycle for an existing ORC geothermal power station and their study revealed that a solar rankine cycle uses less brine than a normal geothermal power station. Calise et al. [7] carried out the simulation of a hybrid of geothermal and solar were their study produce a decrease in carbon dioxide (CO_2_) emissions equivalent to 97.36% and savings on basic energy of 94.54%.

A geothermal solar hybrid system works in a way that when there is no enough sunlight and during the night, it uses the Organic Rankine Cycle (ORC) to generate energy. In contrast to the Rankine cycle, which employs water, the ORC system uses organic fluid that boils at a low temperature. Apart from ORC system, there are several uses for organic working fluids, including in heat pumps and refrigerators. Some of research on heat pumps and refrigeration equipment that uses organic working fluids includes the one for Afshari et al. [8] who investigated the performance of the heat pump and suction pressure of the compressor using different refrigerants such as R22 and R134a through an experiment. Afshari et al. [9] also conducted a study comparing heat pumps and refrigeration equipment using various refrigerants, including R134a. This shows that working fluid selection is crucial in all the aspects utilizing organic working.

For geothermal system, studies have demonstrated that heat from the geothermal brine with a low temperature can be employed to generate power using ORC [1,10]. However, the efficiency of the system is reported to be around 13%. This can be improved by using a geothermal-solar hybrid system. The performance of the ORC is highly influenced by the organic working fluid used. Therefore choosing a working fluid for an ORC power plant should be done with caution. Working fluids are selected based on their health and safety, economic, environmental considerations and thermodynamic properties [11,12]. According to DIPippo [11], the environmental, health, and safety characteristics of prospective working fluids includes flammability and toxicity. It also involves the likelihood that it could cause global warming called Global Warming Potential (GWP) and the potential of the fluid to deplete the ozone called Ozone Depletion Potential (ODP). The organic working fluid, must have GWP less than 150 and should have no ODP [12]. The International agreements including the Paris Agreement, Kyoto Protocol, and Montreal Protocol, insist on the removal of dangerous compounds that contribute to global warming [11,12,13,14]. Majority of previous studies [2,6,15,16] on geothermal solar hybrid cycles have analyzed the effectiveness of the ORC using higher GWP working fluids such as R134a with a GWP of 1430 and R245fa with a GWP of 1030 which possess danger to the environment. The working fluids with lower toxicity are also recommended for use. Refrigerant are grouped in terms of safety according to ASHRAE standard 34 [17].

The working fluids can also be classified into three categories which are isentropic fluids, dry fluids, and wet fluids. This classification is based on the T-s saturation curve's expansion process' slope. Fluids that are wet have a negative slope, fluids that are isentropic have a vertical slope, and fluids that are dry have a positive slope. For usage in ORC, dry working fluids are desirable. Droplets created by the later stages of wet fluid expansion in a turbine impinging on the turbine blades and causing erosion [18,19] and isentropic fluids are phasing out due to their higher Global Warming Potential (GWP) therefore they are not preferred. Another important parameter in selecting the working fluids is the stability temperature. This is crucial to determine the highest temperature at which the power plant can be operated. Decomposition of working fluids normally occurs when a working fluid is exposed to a very high temperature; therefore care should be taken to avoid this as it leads to a reduction in efficiency of the working fluid. According to Rajabloo [20], when subjected to heat, organic working fluid may potentially disintegrate, resulting in a mixture of working fluid.

Different working fluids have been considered for use in ORC and their performance reported. Salman et al. [21] carried out a research to compare the thermal efficiency of n-butane (R600), R236ea, R245fa and n-hexane working fluids using aspen plus software. N-butane outperforms other working fluids with a thermal efficiency of 13.55% at around 70 °C–90 °C. In their analysis, the effect of solar heat fluid on organic working fluid performance was not investigated in addition to the pump power required by the working fluids and the net work output. Furthermore they analyzed R245fa with a higher GWP of 1030. Najjar and Qatramez [10] performed a study using different working fluids for ORC power generation that uses a geothermal temperature source in the range of 200–260 °C. Highest net power output and efficiency of 24.89 MW and 18.76% were obtained in their study using R11 fluid. The selected working fluid R11 has a higher GWP of 4750, hence not suitable for ORC applications.

Nurhilal et al. [22] performed a simulation for the ORC system driven by geothermal in Indonesia using n-pentane. Ashuri et al. [23] performed a study for a small-scale power generation driven by a coupled ORC and a parabolic trough solar collector in Tehran using Thermoflex19 software. In their study the highest net electric efficiency was obtained from benzene. The effect of Solar Heat Transfer Fluid on working fluid performance was not analyzed in their study. Wang et al. [24] used the Aspen Plus software to conduct a comparison of small ORC systems powered by solar employing working fluids R245fa, R134a, and isobutane. Their findings revealed that the conventional system is less suitable for residential applications than the solar system for the small scale using a thermal driven pump.

Wang et al. [25] carried out a thermodynamic economic analysis of a solar powered ORC systems for small scale. Their studies revealed that a model using isobutane in an ORC system outperforms other models in terms of thermodynamic performance. Song et al. [26] also analyzed the thermodynamic and economic of a geothermal ORC systems for power generation. According to their research, low critical temperature working fluids perform better when superheated but higher critical temperatures degrades when superheated. Song et al. [27] in their other study performed the thermodynamic and financial analysis for carbon dioxide-ORC systems for the hybrid of geothermal and solar for electricity generation and their results revealed that hybrid systems are superior.

Dai et al. [28] experimentally performed a research to investigate the temperature at which hexamethyldisiloxane (MM) is stable as a working fluid for ORC. The authors reported that MM started to decompose at the temperatures higher than 240 °C. Khider el al [29]. performed the study through experiment to investigate the performance of hexamethyldisiloxane and alkanes for use in ORC system. In comparison to alkanes, they discovered that hexamethyldisiloxane is a more suitable working fluid in terms of temperature stability. Invernizzi et al. [30] performed the thermal stability of the working fluids in the organic Rankine cycle using n-pentane, cyclo-pentane, and toluene. They found out that at 350 °C, Cyclopentane was thermally stable. The experiment to investigate the temperatures at which decomposition occur for n-butane, toluene, and n-pentane for ORC applications was also carried out by Pasetti et al. [31]. They reported that, Toluene sample was remarkably stable for temperatures of around 400 °C, n-pentane was thermally stable for temperatures below 315 °C and n-butane was thermal stable for temperature close to 290 °C. Since n-butane, n-pentane, Cyclopentane, Hexamethyldisiloxane (MM), and Toluene working fluids have higher thermal stability temperature; it makes them suitable for geothermal solar hybrid applications.

From the reviewed literature, it can be deduced that n-butane (R600), n-pentane, Cyclopentane, Hexamethyldisiloxane (MM), and Toluene working fluids have not been extensively analyzed for geothermal solar hybrid applications. In this study, the aforementioned working fluids are studied based on the net power produced and the needed pump power. Additionally, the effect of Therminol VP-1 flow rate and pressure and geothermal source pressure and flow rate on organic working fluid net power output were analyzed and optimized. A suitable working fluid for the hybrid system was chosen on the basis of its greater net power output. The selected working fluid was used to determine the average hourly power output in each month of the hybrid system using the irradiance of Olkaria II power station.

## 2 Methodology

### 2.1 Process simulation

**Figure 2:**
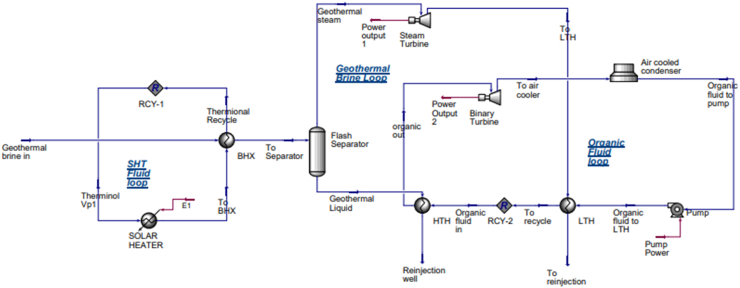
Modeled flash geothermal-solar hybrid in Aspen HYSYS.

The process simulation was performed using Aspen HYSYS V12.1® commercial thermodynamic software. Figure 2 shows the simulated geothermal-solar hybrid power plant diagram. The modeled hybrid system consists of three loops: Solar Heat Transfer (SHT) loop, geothermal brine loop and organic fluid loop. The system's primary components are the heat exchangers, turbines, air condenser, parabolic trough solar collector and the pump. The operation of the system is that, the geothermal waste brine at a pressure, temperature and flow rate of 6 bar, 158.9 °C and 157.2 kg/s is passed through the Brush Heat Exchanger (BHX) where it is getting additional heat from the Therminol VP-1 fluid circulating through the parabolic trough solar collector. The heat input from the brine is given by equation (7) and the solar collector's parabolic trough's heat input is given by equation (8) [32]. When the parabolic trough solar collector heats the brine to higher temperature the total heat of the brine obtained is as illustrated equation (6) [6,15]. The superheated steam from the BHX is separated by the flash separator into the steam and liquid. The steam drives the Steam Turbine (ST) to drive the generator for power. The steam from the Steam turbine is transferred to the Low Temperature Heat Exchanger (LTHE) where the organic fluid is heated and then sent back to the reinjection well. The organic fluid is also heated by the liquid brine extracted from the separator flowing through the High Temperature Heat Exchanger (HTHE). The heated organic fluid produces steam which drives the Binary Turbine (BT) to generate additional power.

### 2.2 General assumptions

The following assumptions were made during the simulation process.

• All the components and processes are considered to be at steady state.
• The heat and pressure losses in all system components and piping are neglected.
• The turbine and pump are adiabatic with 85% isentropic efficiencies.
• The variations in kinetic and potential energy are not taken into account.

Based on the above assumptions, the following is the general steady state energy balance equations [2,23]:

(1) $$\sum {\dot{m}}_{in}=\sum {\dot{m}}_{out}$$

(2) $$Q-W+\sum {\dot{m}}_{in}h_{in}-\sum {\dot{m}}_{out}h_{out}=0$$

Where $\dot{m}\;\left(kg/s\right)$ is the mass flow rate and h (kJ/kg) stand for the specific enthalpy of the system's working fluid streams and Q (W) and W (W) stand for the heat and work energy passing via the component boundaries.

The net work ($W_{net}$), turbine work ($W_{turbine}$) and the pump work ($W_{pump}$) were calculated using the equations as shown below [33]:

(3) $$W_{net}=W_{turbine}{-W}_{pump}$$

(4) $$W_{Turbine}={\dot{m}}_{f}\;\left(\;h_{in}-h_{out}\right)$$

Where ${\dot{m}}_{f}$ is the mass flow rate, $h_{in}$ is the specific enthalpy at the turbine entry and $h_{out}$ is the specific enthalpy at the exit of the turbine.

(5) $$W_{pump}=\frac{\upsilon_{p\;}\left(P_{out}{-P}_{in\;}\right)\times {\dot{m}}_{f}}{\eta_{p}}$$

Where $P_{out}$ and $P_{in}$ insignify, respectively, the pump's outlet and inlet pressure. The efficiency and the fluid's specific volume of the pump are denoted by $\eta_{p}$ and $\upsilon_{p}$, respectively.

The heat into the system ($Q_{in}$), is given by the formula below:

(6) $$Q_{in}=Q_{geo}+Q_{solar}$$

Whereby the geothermal heat ($Q_{geo}$), is given by the following equation.

(7) $$Q_{geo}={\dot{m}}_{geo}\left(h_{FW}-h_{PW}\right)$$

Where $h_{FW}$ and $h_{PW}$ are the enthalpy of the brine to the injection wells and geothermal brine from the separator, respectively, and ${\dot{m}}_{geo}$ is the geothermal brine mass flow rate.

The energy collected by the solar collector ($Q_{solar})$ is governed by equation (8) [32]:

(8) $$Q_{solar}=\tau \alpha IA_{c}-U_{c}A_{c}(T_{s}-T_{amb}$$

Where $Q_{solar}$, τ , α, I, $A_{c}$, $U_{c}$, $T_{s}$ and $T_{amb}$ are the heat energy collected, transmissivity of the cover glass, the absorber plate's absorptivity, the solar irradiance (W/m^2^), the area of the collector (m^2^), collector's thermal loss coefficient (W/°C), storage Theminol VP-1 temperature and outdoor ambient temperature, respectively. The incident solar radiation for Olkaria II power station in this study is obtained from System Advisor Model (SAM) software for the year 2020.

### 2.3 Validation of results and process simulation

**Figure 3:**
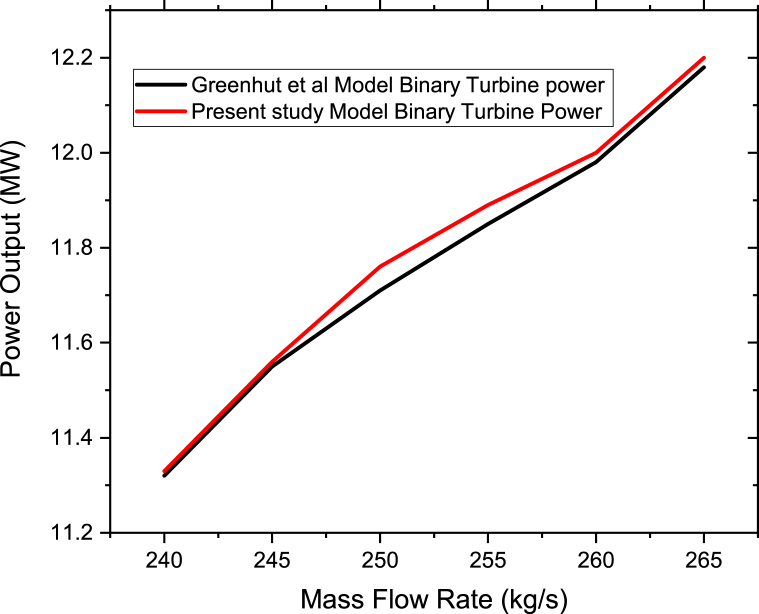
Flash geothermal-solar hybrid model validation.

The generated data from the modeled process simulation of the flash geothermal and solar power plant was validated with results from previous studies of Greenhut et al. [6] as shown in Figure 3. Grenhut et al. [6] compared the flash hybrid and superheat hybrid cycle in Nevanda using R134a working fluid in terms of power output where the Flash hybrid showed the best performance compared to the superheat hybrid. In the present study, the flash hybrid is analyzed using low GWP working fluids for the waste brine at Olkaria II power plant. Further comparisons were made between the findings of the current study and those of Rajabloo [20], Hu [34], Liang and Yu [35], and others. The comparisons in results of the previous studies shows good agreement with the present study as explained in the results and discussion section below.

### 2.4 Selection of the working fluid

In this study, working fluids were chosen using the following criteria: (i) Thermodynamic properties; which are low Normal Boiling Point (NBP), high density and moderate Critical Temperature (CT) and Critical Pressure (CP). Only dry working fluid were considered because droplets produced during the later stages of wet fluid expansion in a turbine impinging on the turbine blades and causing erosion [18,19]. On the other hand, isentropic fluids are phasing out due to their higher GWP. (ii) Health and safety; the selection was based on the ASHRAE standard 34 in which only lower toxicity working fluids were considered. (iii) Cost; working fluids analyzed here are those of reasonable price and easily available. The current market price of the selected working fluid is as tabulated in Table 1. (iv) Thermal stability temperatures; the temperature are tabulated in Table 1. To be able to determine the power plant's maximum operating temperatures, the fluid's thermal stability is essential, this will help to avoid decomposition of working fluids which results in low thermal effectiveness of the system [36]. The analyzed working fluid as shown in Table 1 can be used to replace highly used high GWP working fluids such as R-245fa, R-236ea, R227ea and R134a among others.

**Table 1:** Properties of organic working fluids analyzed using geothermal solar hybrid power cycle [11,[28], [29], [30], [31],^36^,^37^].

Working Fluids	NBP (°C)	CT (°C)	CP (kPa)	ASHRAE SAFETY GROUP	ODP	GWP (100 years)	Expansion	Density (kg/m^3^)	Cost ($/kg or $/ton)	Thermal Stable Temperature (°C)
n-Pentane	36.1	196.5	3364	A3	0	3	dry	620.8	$2500- $2800/ton	315
Toluene	110.6	591.6	4130	A3	0	3.3	dry	867	$1700-2380/ton	400
Hexamethyldisiloxane (MM)	100	245	1940	A2	0	3	dry	258.2	US$5.80/kg	240
n-butane (R600)	−0.5	152	3796	A3	0	3	dry	553	$1.00–$3.40/kg	290
Cyclopentane	49	238.6	4515	A3	0	3	dry	751	$1700.00–$2680.00/ton	275

## 3 Results and discussion

The selected working fluids as tabulated in Table 1 were analyzed based on the pump power required and net power output by heating Therminol VP1 fluid to a maximum temperature of 400 °C and varying the organic working fluid flow rate as well as Therminol VP1 and geothermal brine pressure and mass flow rate. The above mentioned parameters were investigated to understand their effect on the power output. Finally, the working fluid that produces more net power output among n-butane, n-pentane, Hexamethyldisiloxane, Cyclopentane, and Toluene was selected as a suitable working fluid for the hybrid system. The selected fluid was used to determine the average hourly power output in each month of the hybrid system using the irradiance of Olkaria II power station.

### 3.1 Working fluid mass flow rate

**Figure 4:**
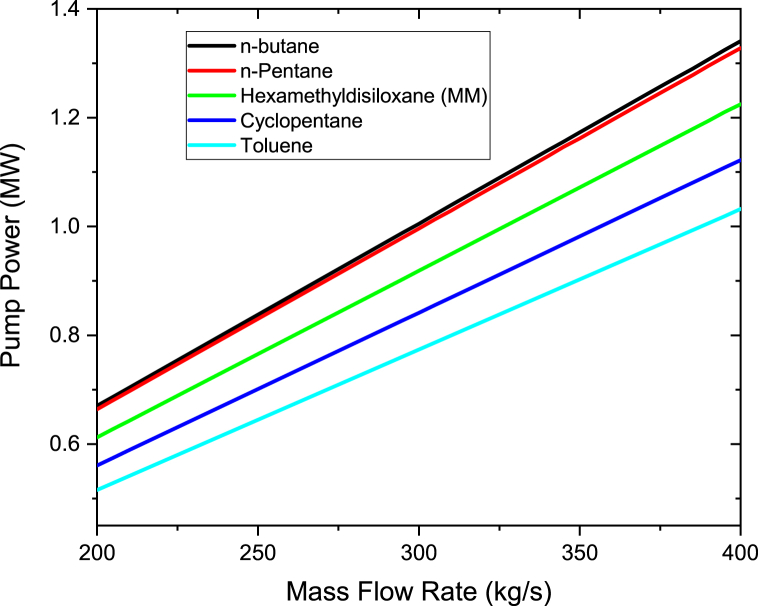
Pump power at different organic working mass flow rates.

To study the effect of the mass flow rate of organic working fluids on the power output, the mass flow rate was varied from 200 to 400 kg/s in steps of 10 kg/s. Therminol VP-1 fluid was heated to a maximum temperature of 400 °C, which was maintained constant when varying the fluid flow rate. As shown in Figure 4, the pump power increases linearly as the working fluids mass flow rate is increased. The highest pump power was noted for n-butane while the lowest for Toluene. This is due to the fact that the pressure at the pump's inlet and outlet for the ORC cycle using n-butane differs significantly from one another (P_out_-P_in_). The working fluid pump must therefore use more energy to be able to pump the required organic working fluid. It can be deduced from equation (5) that working fluids with small pressure difference and/or with low specific volume results in the decrease of pump power. Yang et al. [38] also conducted an investigation on the impacts of different state of condensation on a centrifugal pump with several stages for the ORC and their findings revealed that an increase in pump power is as the result of either a rise in output pressure or a fall in the pump's condensation temperature. Taking n-butane as a reference working fluid, the percentage differences in pump power are 0.925% to n-pentane, 8.64% to Hexamethyldisiloxane (MM), 16.32% to Cyclopentane and 23.1% to Toluene. The results of n-butane, n-pentane, Toluene and MM are in good accord with Borsukiewicz-Gozdur [39] work. Borsukiewicz-Gozdur [39] found that working fluid whose critical temperature is higher have a lower pump power in contrast to whose critical temperature is lower. In this work, the higher values of pump power than the other working fluids are due to its lowest critical temperature as shown in Table 1. However MM pump power is higher than Cyclopentane, this is because of larger difference at the outlet and inlet pressure of the pump of MM compared to Cyclopentane.

**Figure 5:**
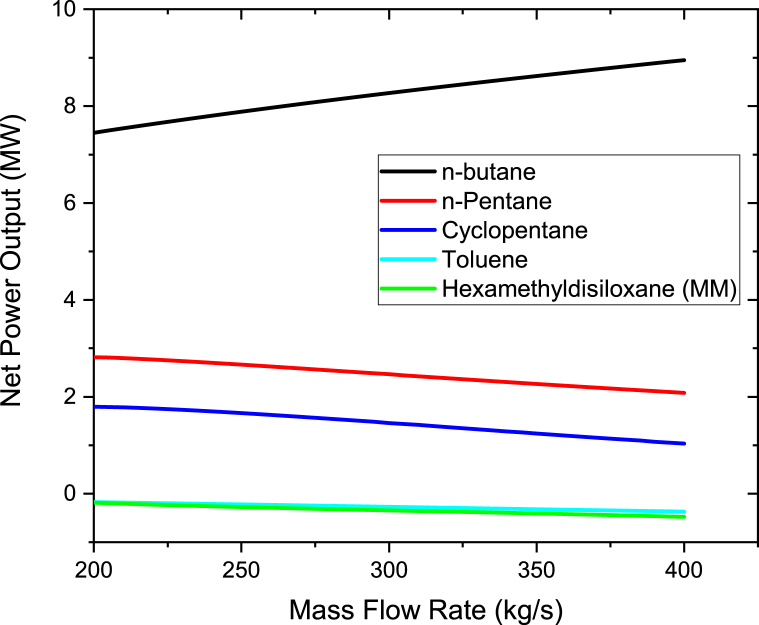
Net power output at different mass flow rates for organic working fluids.

The graph of the net power output and mass flow rate of the working fluid are as shown in Figure 5. The net power outputs were calculated using equations (1),(2),(3),(4),(5) [2,33]. It can be seen that the net power output for n-butane increases as the mass flow rate of the working fluid is increased. Contrary, as the working fluid's mass flow rate was increased, the net power output for Hexamethyldisiloxane (MM), n-pentane, cyclopentane, and toluene declined. This is because for n-pentane, Cyclopentane, Hexamethyldisiloxane (MM), and Toluene, excessive quantity of heat is transmitted from heated geothermal brine to the organic fluid in the heat exchangers as the working fluid's mass flow rate rises. Since the amount of heat is constant, the superheat degree of the vapor steadily decreases when increasing the flow rate of the working fluid. If the working fluid's mass flow rate further ascends, the working fluid at the turbine intake will reach a 2 phase region. Due to this, the turbine's power output decreases. These results are consistent with those of Liang and Yu [35]. Liang and Yu [35] ran an experiment to look into an ORC system employing a scroll turbine for recovering heat from low temperature heat source. Their study found out that as the rate of mass flow of the working fluid rises, the output power and net work increases first and then decline after, this is because of the degree of superheat of the vapor which reduces with a rise in the mass flow rate for the constant heat energy input. Due to this, when the operating fluid is not overheated, at the lower mass flow rates, the greatest net power generation is obtained. Therefore to obtain maximum power output from an ORC system, the system should be regulated to be able to accomplish the lowest superheat intensity at the entry of the turbine. With respect to n-butane, as the working fluid's mass flow rate rises, not too much heat is required to be transferred from hot geothermal brine in the heat exchangers as compared to the other working fluids. The results also show that n-butane produces more net power compared to toluene, n-pentane, cyclopentane, and hexamethyldisiloxane (MM). This is because n-butane has a low critical temperature and a low boiling point as compared to other working fluids as per Table 1. This is due to the fact that the critical temperature of n-butane is near to the temperatures of the saturated steam from the steam turbine and the brine from the flash separator. This results is having a similar trend discovered by Babatunde and Sunday [40] in which they discovered that when the working fluid's critical temperature is close to the temperature from the source of heat, ORC system that uses isentropic or dry working fluids exhibit best results. The other reason for the higher net power output is that since n-butane has a low boiling point as compared to the other organic fluids, this means that flashing occurs at a low temperature when exposed to heat which makes it to produce more net power output.

### 3.2 Effect of therminol VP-1 pressure and mass flow rate

**Figure 6:**
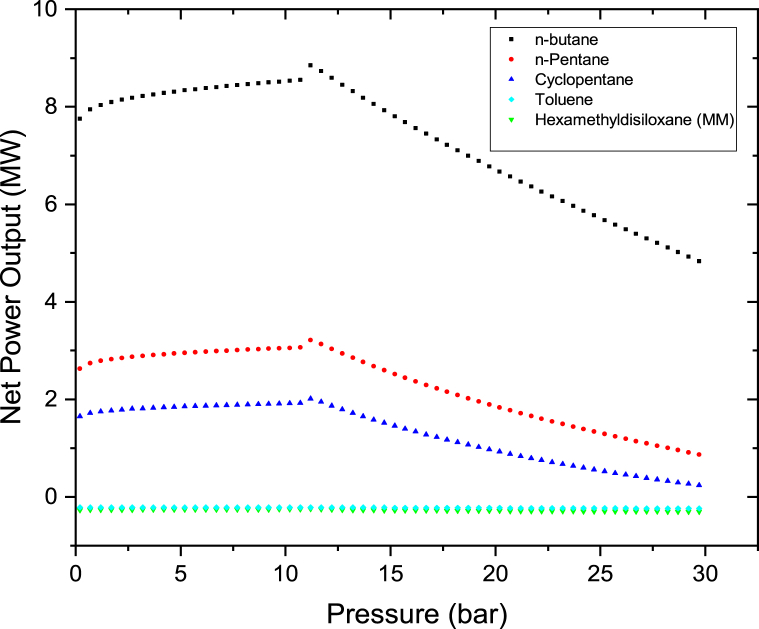
Binary turbine net power output at different Therminol VP-1 pressure for a constant therminol VP-1 mass flow rate of 100 kg/s.

**Figure 7:**
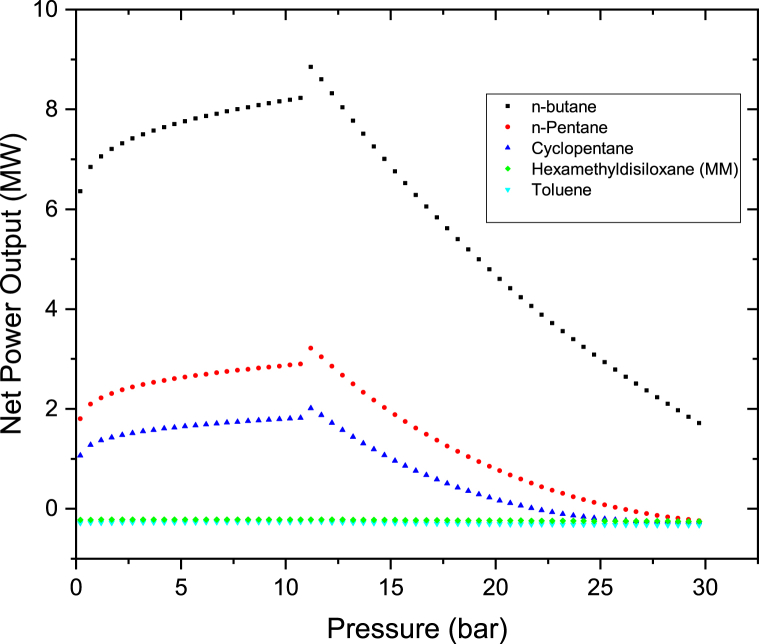
Binary turbine net power output at different Therminol VP-1 pressure for a constant therminol VP-1 mass flow rate of 200 kg/s.

The effects of Therminol VP-1 pressure and rate of flow on the on the power output were investigated by heating Therminol VP-1 fluid to the maximum temperature of 400 °C. First Therminol VP-1 pressure was raised from 0.2 bar to 30 bar in steps of 0.2 bar and thereafter the mass flow rate was increased from 100 kg/s to 200 kg/s. The results as shown in Figures 6 and 7 indicate that the net power rises with varying the pressure from 0.1 bar to 11 bar and thereafter decreases as the pressure is increased to 30 bar. This is because as the Therminol VP-1 is exposed to higher pressures, the vapor's superheat level gradually drops, since less heat is transmitted to the brine via the heat exchanger. As the pressure increases for the same heat source of 400 °C, the degree of superheat decreases since more heat will be required when the pressure is increased. Maximum net power outputs were observed at 11 bar for all the cases. At this point Therminol VP-1 fluid is heated to the optimum amount; hence maximum heat is transferred to the brine via the heat exchanger. To be able to produce maximum power from this hybrid cycle, Therminol VP-1 pressure should be maintained at a pressure of around 11 bar or below. N-butane is leading in net power output in all the cases. Increasing the flow rate of Therminol VP-1 from 100 kg/s to 200 kg/s as illustrated in Figures 6 and 7 leads to a reduction in power output, however the trends of the graphs are the same. This is because the heat is constant from the parabolic trough solar collector and as the flow rate of Therminol VP-1 increases; huge amount of heat is required to be obtained from Therminol VP-1 to the hot brine in the heat exchangers.

**Figure 8:**
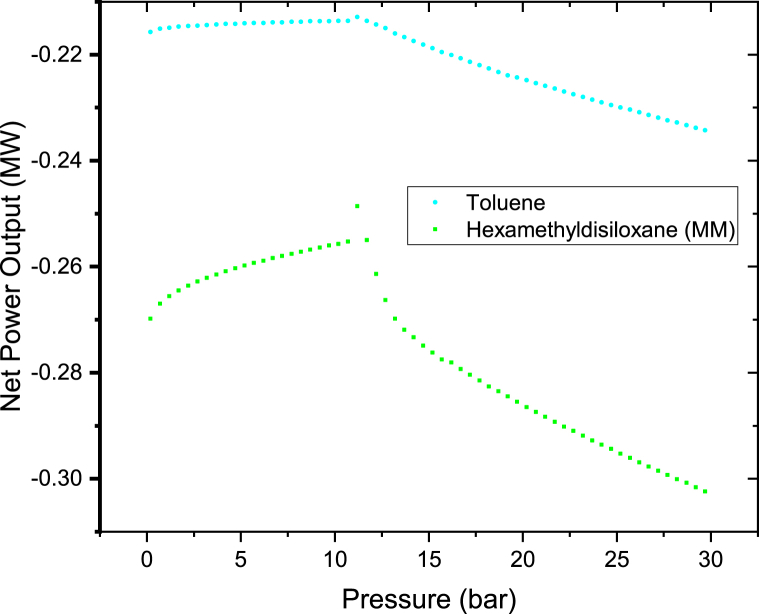
Toluene and MM binary turbine net power output at different therminol VP-1 pressure for a constant therminol VP-1 mass flow rate of 100 kg/s.

**Figure 9:**
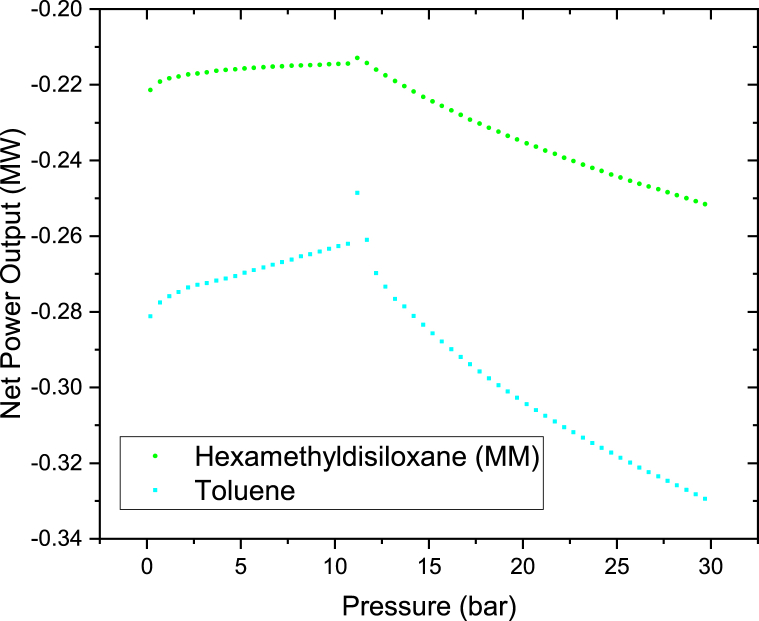
Toluene and MM binary turbine net power output at different therminol VP-1 pressure for a constant 200 kg/s therminol VP-1 mass flow rate.

Since MM and Toluene have very low negative net power output as indicated in Figures 6 and 7. They were further plotted on the separate graphs as shown in Figures 8 and 9. Their results clearly show the same trends as others. The net power outputs are negative because the pump is consuming more power than the power from the binary turbine. Therefore when the net power output was calculated yields the negative value. The net power output is the difference between the pump power and the binary turbine power output as per equation (3). The reason of this very higher pump power is because of the large differential pressure between the pump's outlet and inlet (P_out_-P_in_). This was caused because 240 kg/s higher flow rate of the organic working fluid which was considered in the analysis of this case. Therefore if the rate of flow of the organic working fluid was kept low it will yields in a positive net power output. This is well illustrated in Figure 5 which shows that at low fluid flow rate higher net power output can be produced for n-pentane, Cyclopentane, Hexamethyldisiloxane (MM), and Toluene working fluids of this hybrid system.

**Figure 10:**
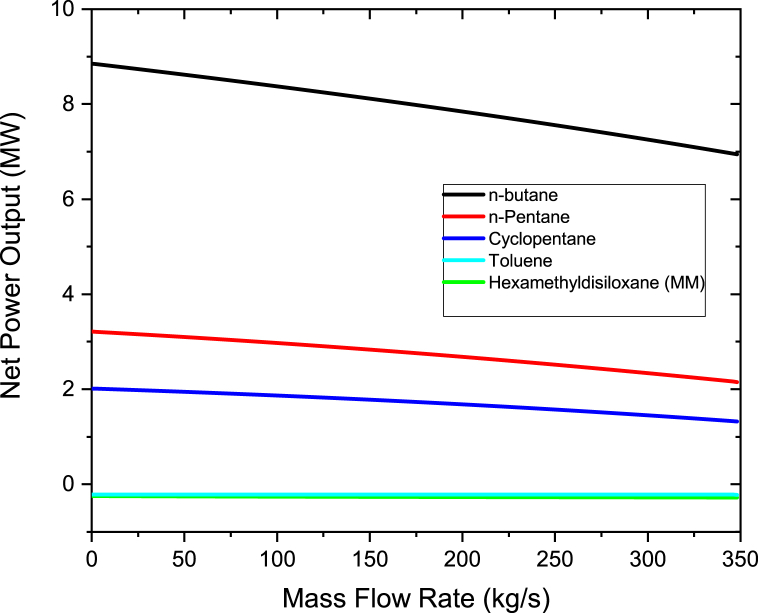
Binary turbine net power output at different therminol VP-1 mass flow rate for a constant therminol VP-1 pressure of 6 bar.

**Figure 11:**
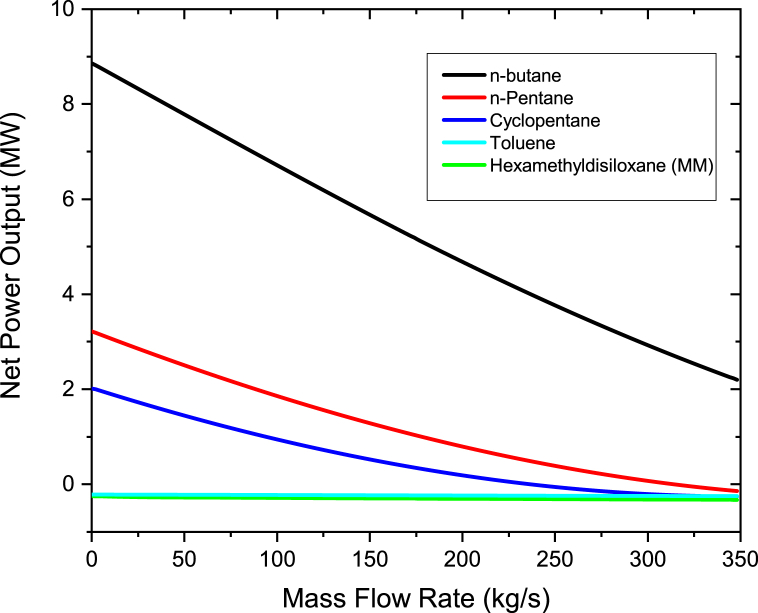
Binary turbine net power output at different therminol VP-1 mass flow rate for a constant therminol VP-1 pressure of 20 bar.

The Therminol VP-1 mass flow rate and pressure were also investigated by heating the Therminol VP-1 fluid to a maximum temperature of 400 °C and increasing the pressure from 6 bar to 20 bar. The range of the mass flow rate was from 0.1 kg/s to 350 kg/s in steps of 2 kg/s. Figures 10 and 11 also show that the binary turbine net power decreases as the Therminol VP1 mass flow rate is increased. As the pressure was raised from 6 bar to 20 bar, the net output power decreases as well. N-butane working fluid still leads the other working fluids in net power output at different solar heat transfer working fluid mass flow rate. These results are in agreement with Alirahmi et al. [41] results, who performed a multigenerational system using solar and geothermal energy to generate energy and provide cooling among other at once. Their results for the effect of the flow rate of Therminol VP-1, Therminol 59 and other solar heat transfer fluids on net power output indicated that increasing the flow rate of solar fluid can significantly reduce the output power.

**Figure 12:**
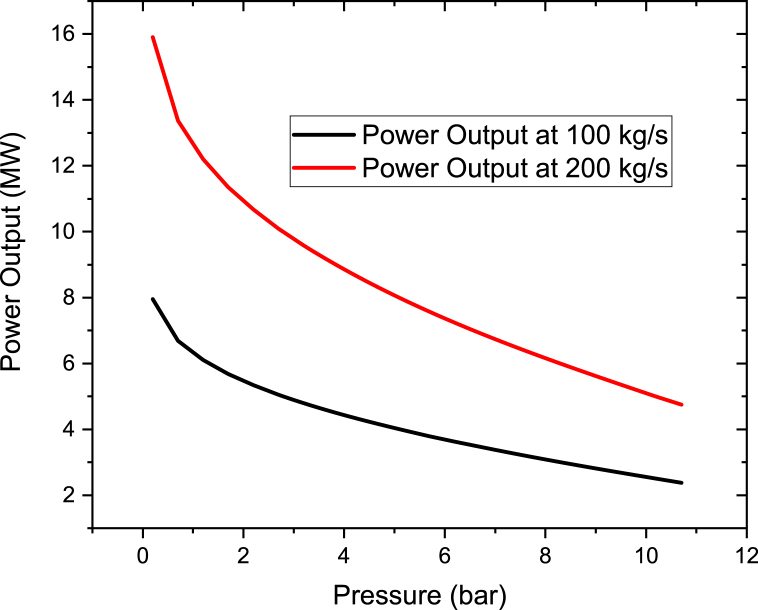
Steam turbine power at different therminol VP-1 pressure for a constant therminol V-1 flow rate of 100 kg/s and 200 kg/s.

The Steam turbine power output was also investigated with respect to the Therminol VP-1 fluid pressure and flow rate. The result is in agreement with the Binary turbine power output. For example as illustrated in Figure 12, as the Therminol VP-1 pressure rises, the steam turbine power declines. The results clearly shows that at low Therminol VP-1 pressure more power can be obtained from the Steam turbine as well.

### 3.3 Effect of geothermal brine pressure

**Figure 13:**
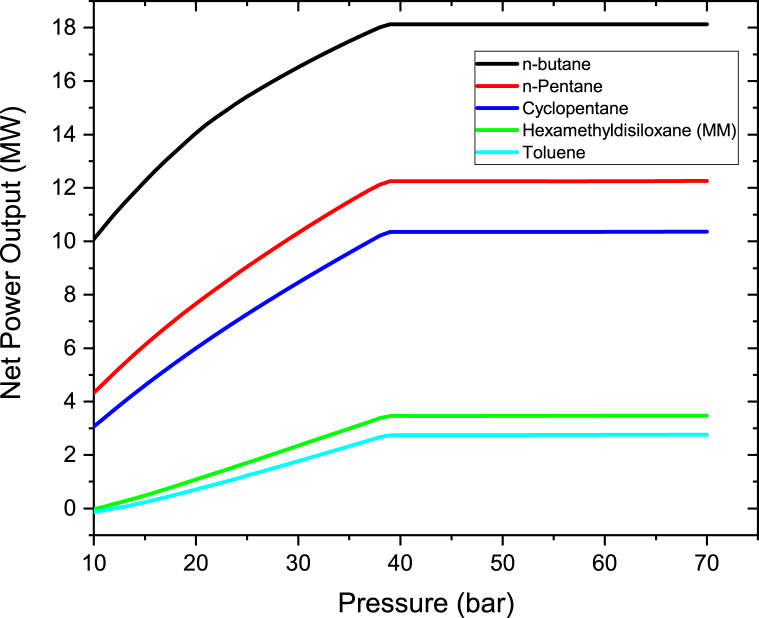
Binary turbine net power output at different geothermal brine pressure.

The brine pressure was varied from 10 bar to 70 bar in 1 bar steps as depicted in Figure 13. The findings show that the Binary turbine power, increases for all the working fluid until a pressure of 39 bar and becomes constant from 39 bar to 70 bar. This is when the brine is exposed to higher pressure, the brine temperature from the BHX heat exchanger increases until it becomes constant. The temperature becomes constant due to evaporation or change of state. The energy that is produced when a change in state takes place is utilized to modify the binding energies instead of increasing the molecules' kinetic energy, therefore the temperature remains constant. The brine will also be able to extract more heat in the BHX heat exchanger. The net power output becomes constant from 70 bar because the temperature is constant. The behavior of the brine when exposed to higher pressure behaves in an opposite way compared to Therminol VP-1 when exposed to higher pressure. This is because the brine is the one extracting heat from Therminol VP-1 fluid through the Heat exchanger.

### 3.4 Effect of geothermal brine mass flow rate

**Figure 14:**
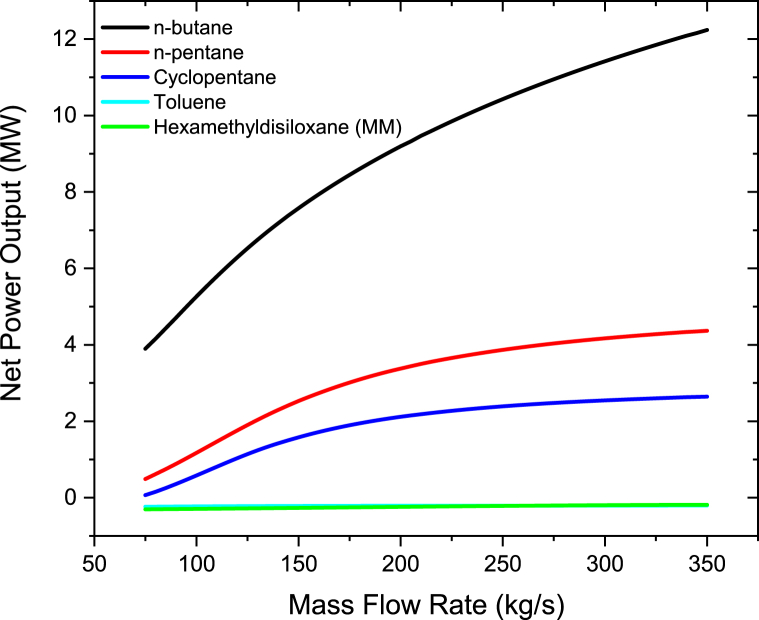
Binary turbine net power output at different geothermal brine mass flow rate.

The brine mass flow rate was varied from 50 kg/s to 350 kg/s in steps of 2 kg/s as indicated in Figure 14. The results show that Binary turbine power increases with increasing brine mass flow rate. This is when the rate of flow of the brine rises; the brine extracts more heat through the BHX heat exchanger. As a result, the heat exchanger allows the working fluids to extract more heat, increasing the power output. In comparison to Therminol VP-1, the behavior of brine when its mass flow rate increases behaves in an opposite way as well. This means that more power can be obtained as the rate of brine mass flow increases. These findings concur with findings of Altun et al. [42]. Altun et al. [42] thermodynamically investigated an operational geothermal ORC power plant using the measured data from the AFJET company. Their results showed that the amount of turbine work increases noticeably as the brine mass flow rate rises.

### 3.5 Irradiance and monthly hourly power output

**Figure 15:**
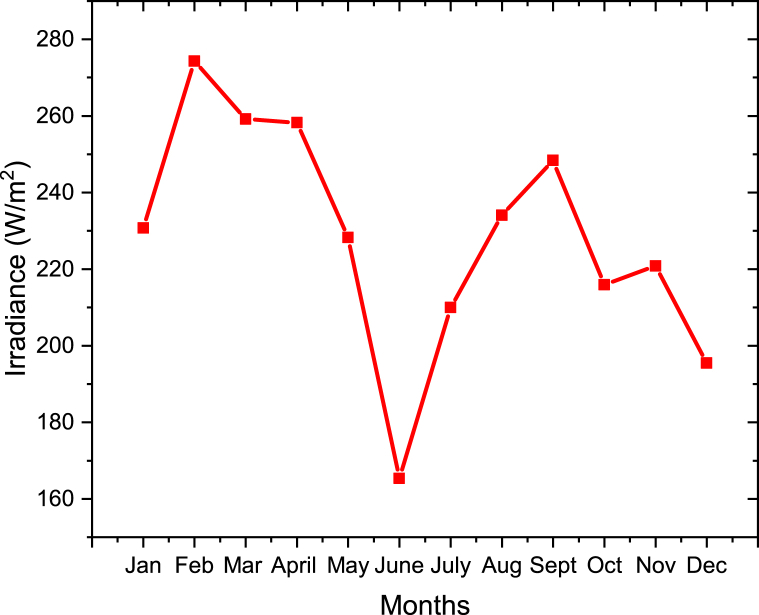
Average hourly Irradiance in each month for Olkaria II power plant.

The average hourly irradiances in each month were obtained from the System Advisor Model (SAM) Software as shown in Figure 15. The irradiances used were for the year 2020. The results showed that the month of February has the maximum average hourly irradiance of 274.3 W/m^2^ and the month of June has the minimum average hourly irradiance of 165.4 W/m^2^ compared to the other months as shown in Figure 15. This is because in February is during summer and in June is during winter. During summer is very hot, therefore the irradiance is higher and during winter is cold therefore the irradiance is lower.

**Figure 16:**
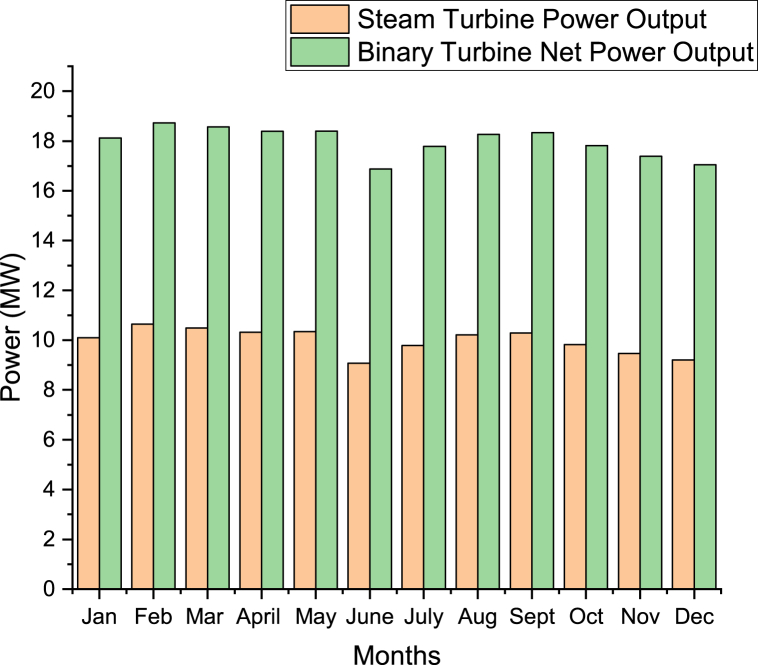
Average hourly power output in each month for the hybrid cycle.

Figure 16 shows the average hourly power output in each month which was obtained using the irradiances in Figure 15. This was performed using the selected n-butane working fluid. The power output was performed using Aspen HYSYS software. February has maximum average hourly power output where the Steam turbine has an average daily power output of 10.48 MW and Binary turbine net power output of 18.56 MW as compared to other months. June has minimum average hourly power output where the steam turbine has an average hourly power output of 9.07 MW and binary turbine net power output of 16.88 MW as compared to other months.

**Figure 17:**
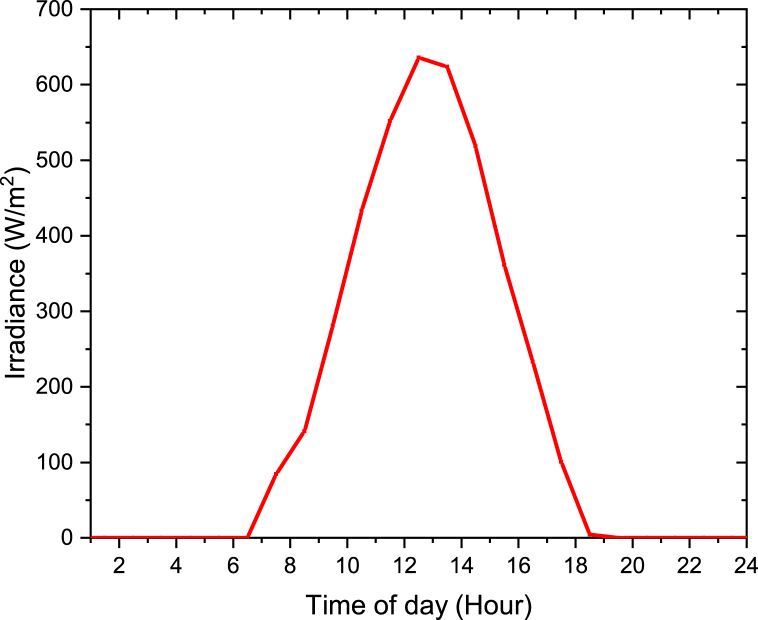
Irradiance for June, 6th at Olkaria II power station.

**Figure 18:**
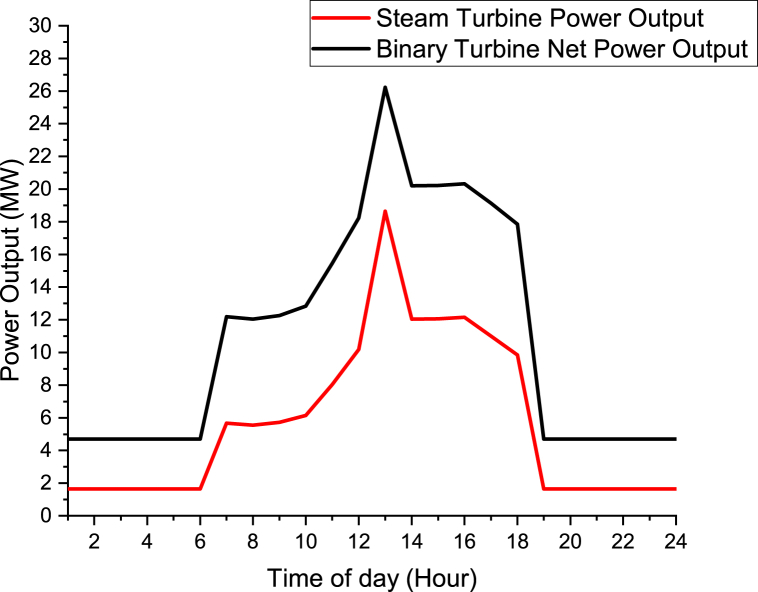
Power Output for June, 6th for the geothermal-solar hybrid power plant.

June 6th day was selected to be analyzed in this study as shown in Figures 17 and 18. It was chosen to be able to understand the trend of the irradiance and output output power during one of the day. As shown in Figure 17, the irradiance is 0 W/m^2^ as from 07:00 p.m. to 06:00 a.m. Therefore at this time the hybrid power plant will only be working on the geothermal source inputs without the solar collector loop. The irradiance is zero since is during the night and there is no sun rays. From 07:00 a.m. to 06:00 p.m. the hybrid cycle will be fully operating. That is during the day. The hours with more irradiance are from 11:00 a.m. to 15 p.m. The maximum irradiance of 636 W/m^2^ was obtained at 1:00 p.m. During 1:00 p.m. is the hottest time of the day thus maximum irradiance was obtained.

As shown in Figure 18, the power output for June, 6th is as shown. From 07:00 p.m to 06:00 a.m, the Steam turbine has the same power output of 1.65 MW and Binary turbine has the same power of 4.71 MW. Both Binary turbine and Steam turbine power are constant for that period because at that period the heat is only from the geothermal source and the solar collector is not functional because there is no irradiance. From 07:00 a.m. to 06:00 p.m. the solar collector will also be in operational hence maximum power will be obtained. The maximum power was obtained at 1:00 p.m. with Steam turbine power output of 18.64 MW and Binary turbine net power output of 26.23 MW.

## 4 Conclusion

Five working fluids that have no potential to deplete the ozone or cause global warming have been examined to determine the most appropriate fluid to operate a hybrid geothermal-solar Organic Rankine Cycle. From the findings the following can be concluded: N-butane is leading in net power as compared to n-Pentane, Cyclopentane, Hexamethyldisiloxane (MM), and Toluene working fluids. Based on these, n-butane (R600) was selected as the suitable working fluid to operate a flash hybrid of geothermal and solar power plant at Olkaria II in Kenya. In order to obtain maximum power output from this hybrid system, Therminol VP-1 needs to be kept at a pressure equal to or lower than 11 bar and the flow rate should be kept low close to 0.2 kg/s. Furthermore, the brine pressure and flow rate behave in an opposite way as the Therminol VP-1 pressure and flow rate. Which means the brine can produce more power output at higher pressure and mass flow rate. With n-butane as the chosen working fluid, the power output is highest in February and lowest in June.

## Nomenclature

### Subscripts and abbreviations

ORC: Organic Rankine Cycle
ASHRAE: American Society of Heating, Refrigerating and Air-Conditioning Engineers
HTHE: High Temperature Heat Exchanger
LTHE: Low Temperature Heat exchanger
BHX: Brush Heat Exchanger
PTSC: Parabolic Trough Solar Collector
ODP: Ozone Depletion Potential
GWP: Global Warming Potential
SAM: System Advisor Model
SHT: Solar Heat Transfer
in: inlet
out: outlet
geo: geothermal
sol: solar
c: collector
amb: ambient
s: storage
NBP: Normal Boiling Point
CT: Critical Temperature
CP: Critical Pressure
IW: Injection well
PW: Production well
p: pump
f: fluid

### Variables

m: Mass flow rate (kg/s)
$\dot{Q}$: Heat input (W)
W: Work output (W)
h: Enthalpy (kJ/kg)
P: Pressure (pa)
I: Incident solar radiation (W/m^2^)
A: Area (m^2^)
T: Temperature (°C)
U: Thermal loss coefficient

### Greek letters

τ: Transmissivity
α: Absorptivity
η: Efficiency
